# Pseudotumoral form of granulomatosis with polyangiitis

**DOI:** 10.11604/pamj.2017.26.173.12195

**Published:** 2017-03-27

**Authors:** Fadwa Mekouar, Naoual El Omri

**Affiliations:** 1 Internal Medicine Department, Mohammed V Military Teaching Hospital, Rabat, Morocco

**Keywords:** Cough, granulomatosis with polyangiitis, ANCA

## Image in medicine

A 65-year-old woman, non-smoker presented with a two-month history of cough. She also complained of arthralgia and weight loss. The clinical examination revealed a febrile patient. The chest radiograph and the scanner revealed right upper lobar mass, with spiculated contours (A, B). Biology showed an inflammatory syndrome. Polymerase chain reaction for Mycobacterium tuberculosis and tumor markers was negative. A lung biopsy showed non caseous granulomatous vasculitis. The cytoplasmic antineutrophil cytoplasmic antibody (cANCA/PR3/ANCA) showed a positive result; Wegener's granulomatosis was finally confirmed. The patient was treated with prednisone and cyclophosphamide with favorable outcome. A follow-up chest radiography two weeks after treatment showed resolving pulmonary lesions (C).

**Figure 1:**
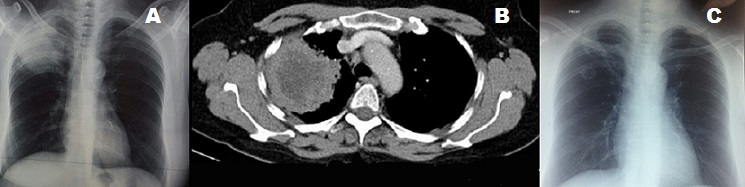
A) chest radiography revealed right upper lobar mass, with spiculated contour; B) chest scanner revealed right upper lobar mass, with spiculated contours; C) chest radiography two weeks after treatment showed resolving pulmonary lesions

